# A randomized study of the immunogenicity and safety of Japanese Encephalitis Chimeric Virus Vaccine (JE-CV) in comparison with SA14-14-2 Vaccine in children in the Republic of Korea

**DOI:** 10.4161/hv.29743

**Published:** 2014-11-06

**Authors:** Dong Soo Kim, Guy Houillon, Gwang Cheon Jang, Sung-Ho Cha, Soo-Han Choi, Jin Lee, Hwang Min Kim, Ji Hong Kim, Jin Han Kang, Jong-Hyun Kim, Ki Hwan Kim, Hee Soo Kim, Joon Bang, Zulaikha Naimi, Valérie Bosch-Castells, Mark Boaz, Alain Bouckenooghe

**Affiliations:** 1 Yonsei University College of Medicine; Severance Children's Hospital; Seoul, Korea; 2 Sanofi Pasteur, Lyon, France; 3 National Health Insurance Service Ilsan Hospital; Gyeonggi-do, Korea; 4 KyungHee University Hospital; Seoul, Korea; 5 KEPCO Medical Center; Seoul, Korea; 6 Yonsei University Wonju College of Medicine; Kangwon-do, Korea; 7 Gangnam Severance Hospital; Seoul, Korea; 8 The Catholic University of Korea Seoul St. Mary's Hospital; Seoul, Korea; 9 The Catholic University of Korea St. Vincent's Hospital; Gyeonggi-do, Korea; 10 Sanofi Pasteur; Seoul, Korea; 11 Sanofi Pasteur; Marcy l’Etoile, France; 12 Global Clinical Immunology; Sanofi Pasteur; Swiftwater, PA USA; 13 Sanofi Pasteur; Singapore

**Keywords:** Japanese encephalitis (JE) vaccine, Phase 3 trial, children, immunogenicity, safety

## Abstract

A new live attenuated Japanese encephalitis chimeric virus vaccine (JE-CV) has been developed based on innovative technology to give protection against JE with an improved immunogenicity and safety profile. In this phase 3, observer-blind study, 274 children aged 12−24 months were randomized 1:1 to receive one dose of JE-CV (Group JE-CV) or the SA14–14–2 vaccine currently used to vaccinate against JE in the Republic of Korea (Group SA14–14–2). JE neutralizing antibody titers were assessed using PRNT_50_ before and 28 days after vaccination. The primary endpoint of non-inferiority of seroconversion rates on D28 was demonstrated in the Per Protocol analysis set as the difference between Group JE-CV and Group SA14–14–2 was 0.9 percentage points (95% confidence interval [CI]: −2.35; 4.68), which was above the required −10%. Seroconversion and seroprotection rates 28 days after administration of a single vaccine dose were 100% in Group JE-CV and 99.1% in Group SA14–14–2; all children except one (Group SA14–14–2) were seroprotected. Geometric mean titers (GMTs) increased in both groups from D0 to D28; GM of titer ratios were slightly higher in Group JE-CV (182 [95% CI: 131; 251]) than Group SA14–14–2 (116 [95% CI: 85.5, 157]). A single dose of JE-CV was well tolerated and no safety concerns were identified. In conclusion, a single dose of JE-CV or SA14–14–2 vaccine elicited a comparable immune response with a good safety profile. Results obtained in healthy Korean children aged 12−24 months vaccinated with JE-CV are consistent with those obtained in previous studies conducted with JE-CV in toddlers.

## Abbreviations

AE: adverse event
AESI: AE of Special Interest
AR: adverse reaction
CI: confidence interval
FAS: Full Analysis Set
GMT: Geometric mean titers
GMTRs: GM of titer ratios
JE: Japanese encephalitis
JE-CV: JE chimeric virus vaccine
JEV: JE virus
MBDV: mouse brain derived inactivated anti-JE vaccines
PP: Per Protocol
PRNT_50_: 50% plaque reduction neutralization test
SAE: serious adverse events.

## Introduction

Japanese encephalitis (JE) is a mosquito-borne, vaccine preventable viral disease that is seasonally endemic or epidemic in nearly every country in Asia.^1,2^ JE virus (JEV) is the most important cause of viral encephalitis in Asia.^3^ JEV is estimated to be responsible for at least 50,000 cases of clinically manifest disease per year, mostly among children aged less than 10 y, and is recognized as a significant cause of childhood morbidity and mortality. However, these figures are thought to considerably underestimate the true burden of JE, given inadequate JE surveillance and reporting in most countries of the Southeast Asia and western Pacific region.^4,5^

In the Republic of Korea, a live attenuated JE vaccine SA14–14–2 (CD.JEVAX®, Chengdu Institute of Biological Products, People's Republic of China) is one of the currently available products used to vaccinate against JE. Inactivated JE vaccines are also available in the country. The recommended schedule of SA14–14–2 vaccine in Korea is a single dose for primary immunization at 12 mo of age and a booster dose one year later. A comprehensive vaccination program has contributed to an estimated JE vaccination coverage (≥ 2 doses) of 75.8% among children aged 7−83 mo.^6^

A new live attenuated JE chimeric virus vaccine (JE-CV) has been developed as an alternative to give protection against JE with an improved overall safety profile compared with the older generation of mouse brain derived inactivated anti-JE vaccines (MBDV). JE-CV was developed using ChimeriVax technology by replacing the genes for yellow fever vaccine (YFV 17D 204) premembrane (prM) and envelope (E) proteins with those of JE attenuated virus strain SA14–14–2.^7,8,9^ This vaccine has been shown to be safe and immunogenic in clinical trials in adults in Australia and the USA^10^ and in toddlers and children in Thailand, the Philippines and Taiwan.^11-14^ A phase 3 study conducted in 300 children aged 9−18 mo in Thailand showed that a single dose of JE-CV or SA14–14–2 vaccine elicited a comparable immune response with a satisfactory safety profile.^14^ The current recommended schedule in the pediatric population is a primary injection from 12 mo of age and over followed by a booster dose between 12 and 24 mo later.^13^ JE-CV was first licensed in Thailand and Australia in 2010.

This was a phase 3 study in children aged 12 to 24 mo in the Republic of Korea. The primary objective was to demonstrate the non-inferiority of JE-CV single dose administration compared with SA14–14–2 vaccine single dose administration, based on the percentage of seroconversion in a JE-CV 50% plaque reduction neutralization test (PRNT_50_) 28 d post-vaccination. Secondary objectives were to further describe the immunogenicity results in both vaccine groups (before and 28 d after the vaccination), safety in all children up to 28 d after vaccination and serious adverse events (SAEs) within 6 mo after vaccination.

## Results

### Participants and demography

The study was conducted between July 2011 and March 2013. As the JE vaccine is generally preferred to be given in Spring and Summer, children were recruited in two cohorts (July to December 2011 and May to September 2012). Fewer children than planned (274 rather than 300) participated in the study owing to slow recruitment at the end of the study (outside the usual JE vaccination period), the expiry date of the comparator and the start of the seasonal influenza vaccination campaign at the end of September (administration of a concomitant vaccine was an exclusion criteria). Though the influenza vaccination rate of toddlers 12−24 mo of age is not available, according to the nationwide vaccine coverage level: Conceptual Methodology and Survey (2008.05.16−2009.03.15), influenza vaccine presented about 50% coverage rate in children 24 mo−6 y of age. Children 6–59 mo of age are the priority target group for influenza vaccination and are recommended to be vaccinated annually in Korea. Children aged 6 mo through 8 y receiving influenza vaccine for the first time should receive two doses administered at least 1 mo apart. Therefore, any children who presented themselves to their healthcare provider during the influenza season would have been recommended to receive an influenza vaccine, and once vaccinated these children could no longer be considered for this study.

Demographic characteristics at baseline were similar between the two vaccine groups (**Table 1**). The mean age was 14.2 mo (range 11.8−22.4 mo). There were slightly more male than female children in both groups.

**Table 1.** Demographic characteristics at baseline (FAS)

	JE-CV (n = 137)	SA14–14—2 (n = 137)	All (n = 274)
Gender, n (%)			
Male	79 (57.7)	80 (58.4)	159 (58.0)
Female	58 (42.3)	57 (41.6)	115 (42.0)
Age, months			
mean (SD) min, max	14.0 (2.0) 11.8, 22.0	14.3 (2.2) 12.1, 22.4	14.2 (2.1) 11.8, 22.4
Body mass index, mean (SD) kg/m^2^	16.8 (1.9)	16.6 (1.8)	16.7 (1.8)

FAS, full analysis set; N, total number of children; n, number of children; SD, standard deviation, min, minimum; max, maximum.

**Figure 1.**
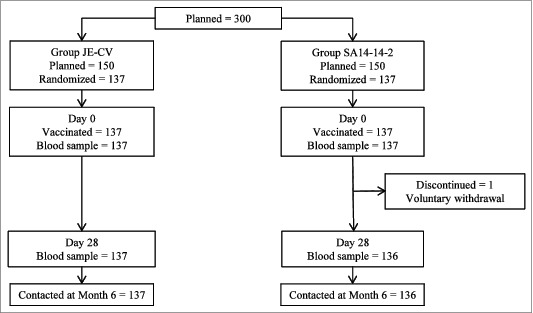
Disposition of children.^1^ JE neutralizing antibody titer ≥ 10 (1/dil) in children who were seronegative (titer < 10 [1/dil])at baseline or a ≥ 4-fold rise in JE neutralizing antibody titers in children who were seropositive (titer ≥ 10 [1/dil]) at baseline;^2^ JE neutralizing antibody titer ≥ 10 (1/dil).

All enrolled children were vaccinated and all except one (in Group SA14–14–2) completed the study (**Fig. 1**). Therefore, the Full Analysis Set (FAS) included 137 children in each group. The Per Protocol (PP) set comprised 119 and 117 children for Group JE-CV and Group SA14–14–2, respectively; main reasons for exclusion were seropositivity against JE-CV at baseline (nine and 10 children in Group JE-CV and Group SA14–14–2, respectively) and post-vaccination blood samples taken outside the protocol-specified time windows (three and seven children in Group JE-CV and Group SA14–14–2, respectively).

### Antibody response to JE-CV 28 d after vaccination

The primary objective of non-inferiority of seroconversion rates 28 d after administration of one dose of JE-CV compared with one dose of SA14–14–2 vaccine was fulfilled as the lower limit of the two-sided 95% confidence interval (CI) of the difference between seroconversion rates in both groups was above –10% (PP set, **Table 2**). All children except one (in Group SA14–14–2) had seroconverted at 28 d after vaccination (PP set, **Table 2**). Seroprotection rates were identical to seroconversion rates since all children in the PP set had a pre-vaccination titer < 10 (1/dil).

**Table 2.** Seroconversion rate 28 d after vaccination (PP set)

	JE-CV	SA14–14–2	JE-CV *minus* SA14–14–2
	**(n = 119)**	**(n = 117)**	
n/M	119/119	116/117	
% (95% CI)	100 (96.9; 100)	99.1 (95.3; 100)	0.9 (−2.35; 4.68)

JE neutralizing antibody titers were measured by a PRNT_50_ test done in Vero cells using JE-CV as the challenge virus. Seroconversion was defined as a JE neutralizing antibody titer ≥ 10 in children who were seronegative at baseline (titer < 10); PP, Per Protocol; N, total number of children; n, number of children; M, number of children with available data; CI, confidence interval; PRNT_50_, 50% plaque reduction neutralization test.

For the FAS, all except one child in each group had seroconverted 28 d after vaccination. Identical seroconversion rates (99.3% [95% CI: 96.0; 100]) were observed in the two vaccine groups. One child in Group JE-CV had a high Day (D) 0 antibody titer (160 [1/dil]) that was similar post-vaccination on D28 (80 [1/dil]); therefore, the child remained seroprotected but the response did not show the ≥ 4-fold increase required for serovonversion. One child in Group SA14–14–2 remained seronegative at D0 and D28. Seroprotection rates were 100% (95% CI: 97.3; 100) and 99.3% (95% CI: 96.0; 100) in Group JE-CV and Group SA14–14–2, respectively.

Geometric mean titers (GMTs) increased in both groups from pre-vaccination to 28 d post-vaccination (PP set). GMTs 28 d after vaccination were 908 (1/dil) (95% CI: 656; 1,256) in Group JE-CV and 579 (1/dil) (95% CI: 427; 784) in Group SA14–14–2. GM of titer ratios (GMTRs) were slightly higher in Group JE-CV (182 [95% CI: 131; 251]) compared with Group SA14–14–2 (116 [95% CI: 85.5; 157]). Similar results were seen in the FAS. GMTs 28 d after vaccination were 1,014 (1/dil) (95% CI: 748; 1,376) in Group JE-CV and 561 (1/dil) (95% CI: 423; 743) in Group SA14–14–2. GMTRs were 181 (95% CI: 133; 247) for Group JE-CV and 99.2 (95% CI: 74.2; 133) for Group SA14–14–2. In the FAS, nine subjects (6.6%) in Group JE-CV and 10 subjects (7.3%) in Group SA14–14–2 had a seroprotective antibody titer (≥ 10 [1/dil]) before vaccination. GMTs were low in both groups, i.e., 5.59 (1/dil) (95% CI: 5.16; 6.05) in Group JE-CV and 5.65 (1/dil) (95% CI: 5.22; 6.11) in Group SA14–14–2.

There were no clinically significant differences in terms of seroprotection or seroconversion seen between the different enrolment centers.

### Safety

Safety data are summarized in **Table 3**. No deaths were recorded during the study and there were no immediate adverse events (AEs) or withdrawals owing to AEs. Throughout the study (D0 to M6), 41 SAEs were reported and split to 20 SAEs in 17 children (12.4%) in Group JE-CV and 21 SAEs in 18 children (13.1%) in Group SA14–14–2, including two AEs of fever that were considered as serious due to the hospitalization of the children; these two AEs were assessed as related to the vaccine by the investigator, both cases were treated and the children continued in the study. All other SAEs were not related to vaccination. AEs of special interest (AESIs) were reported within 28 d after the vaccination by four children in Group JE-CV and seven children in Group SA14–14–2. The most commonly experienced AESI was mild or moderate urticaria affecting one child in Group JE-CV and four children in Group SA14–14–2; only two of these AESIs (one child in each group) were considered by the investigator to be related to vaccination. Other AESIs were mild rash (two children in each group) and allergic rhinitis (one child in Group JE-CV); these were not related to vaccination. One child (23 mo old boy in Group SA14–14–2) experienced an SAE of febrile convulsion in an infectious context 26 d after vaccination; this SAE was not related to vaccination and the child recovered fully and continued in the study.

**Table 3.** Safety overview (safety analysis set)

	JE-CV (n = 137)			SA14–14—2 (n = 137)		
**Children experiencing at least one:**	**n**	**%**	**(95% CI)**	**n**	**%**	**(95% CI)**
**Immediate unsolicited AE**	0	0.0	(0.0; 2.7)	0	0.0	(0.0; 2.7)
**Solicited reaction**	87	63.5	(54.9; 71.6)	97	70.8	(62.4; 78.3)
Solicited injection site reaction	45	32.8	(25.1; 41.4)	56	40.9	(32.6; 49.6)
Pain	35	25.5	(18.5; 33.7)	38	27.7	(20.4; 36.0)
Erythema	23	16.8	(11.0; 24.1)	33	24.1	(17.2; 32.1)
Swelling	6	4.4	(1.6; 9.3)	10	7.3	(3.6; 13.0)
Solicited systemic reaction	72	52.6	(43.9; 61.1)	73	53.3	(44.6; 61.9)
Appetite loss	38	27.7	(20.4; 36.0)	40	29.2	(21.7; 37.6)
Irritability	31	22.6	(15.9; 30.6)	36	26.3	(19.1; 34.5)
Crying abnormal	27	19.7	(13.4; 27.4)	35	25.5	(18.5; 33.7)
Fever	33	24.6	(17.6; 32.8)	34	25.0	(18.0; 33.1)
Drowsiness	23	16.8	(11.0; 24.1)	33	24.1	(17.2; 32.1)
Vomiting	9	6.6	(3.0; 12.1)	14	10.2	(5.7; 16.6)
**Unsolicited AE**	95	69.3	(60.9; 76.9)	99	72.3	(64.0; 79.6)
**Unsolicited AR**	7	5.1	(2.1; 10.2)	8	5.8	(2.6; 11.2)
**AE leading to study discontinuation**	0	0.0	(0.0; 2.7)	0	0.0	(0.0; 2.7)
**SAE during the study**	17	12.4	(7.4; 19.1)	18	13.1	(8.0; 20.0)

Solicited injection site reactions (pain, erythema and swelling) include date within 7 d of vaccination; solicited systemic reactions (fever, vomiting, crying [abnormal], drowsiness, appetite lost and irritability) include data within 14 d of vaccination; N, total number of children; n, number of children; CI, confidence interval; AE, adverse event; AR, adverse reaction; SAE, serious adverse event.

At least one solicited injection site reaction was experienced by 32.8% of children in Group JE-CV and 40.9% of children in Group SA14–14–2. Children experienced injection site pain and erythema more frequently than swelling. Most events were mild, occurred within 3 d after vaccination and lasted for no more than 3 d. One child in Group SA14–14–2 experienced severe injection site pain for 2 d following vaccination; the event resolved spontaneously.

At least one solicited systemic reaction was experienced by a similar percentage of children in Group JE-CV (52.6%) and Group SA14–14–2 (53.3%). The most frequently reported solicited systemic reaction was loss of appetite, affecting 27.7% children in Group JE-CV and 29.2% children in Group SA14–14–2. Other frequently reported solicited systemic reactions were fever, irritability, abnormal crying and drowsiness. Most reactions were mild or moderate, occurred within 3 d after vaccination (except fever, which occurred mainly during D4−D14 in Group SA14–14–2) and lasted no more than 3 d. Six children in each group (4.4%) experienced at least one severe solicited systemic reaction within 14 d of vaccination. These events were rated as severe for no more than 3 d, except one case of vomiting in a child in Group SA14–14–2 that was considered to be severe for 6 d. All events resolved within 14 d after vaccination, except vomiting associated with SAEs of acute gastroenteritis and intussusception (not related to vaccination) experienced by one child in Group JE-CV; this event was unrelated to vaccination and resolved on Day 17.

Unsolicited AEs were reported within 28 d after the vaccination by 95 children in Group JE-CV and 99 children in Group SA14–14–2. These events were mainly diseases of childhood (nasopharyngitis, diarrhea, fever and rhinorrhea); the most frequently reported unsolicited non-serious AEs were classified as ‘infections and infestations’ (mainly nasopharyngitis), experienced by 62.0% of children in Group JE-CV and 54.7% of children in Group SA14–14–2. Unsolicited AEs were mostly mild or moderate, occurred after D8 and resolved within 8 d. Severe unsolicited non-serious AEs were experienced by three children in Group JE-CV (laryngitis, nasopharyngitis and pharyngotonsillitis in one child each) and five children in Group SA14–14–2 (fever in two children; herpangina, pharyngotonsillitis and excoriation in one child each); none was related to vaccination.

Unsolicited adverse reactions (ARs, i.e., vaccine-related in the opinion of the investigator) were reported for seven children in Group JE-CV (contusion in three children; nasopharyngitis in two children; upper respiratory tract infection and urticaria in one child each) and eight children in Group SA14–14–2 (pyrexia in two children; acute tonsillitis, nasopharyngitis, pharyngitis, contusion or bruise at the injection site, rhinorrhoea and urticaria in one child each). All unsolicited ARs were mild or moderate, most occurred within 3 d of vaccination and resolved within less than 8 d.

## Discussion

As part of the development of a new JE vaccine, the World Health Organization (WHO) experts recommend a head-to-head comparison with a vaccine accepted by the regulatory agency of the county where the vaccine is being tested, with a study designed to measure non-inferiority in terms of percentage of seroconversion.^15^ The WHO recommends the use of a serological correlate of protection based on neutralizing antibodies for the evaluation and licensure of new JE vaccines;^15^ a PRNT_50_ titer of 10 (1/dil) is considered as the threshold of acceptance.^4,16^ Our study was designed to follow these recommendations.

It was planned to enroll 150 children per group in this study, in order to have 129 evaluable children in each group. However, 137 children were enrolled per group and the number of children evaluable in the PP set was slightly lower than expected. This did not impact on the power of the study since all children except one in Group SA14–14–2 had seroconverted in the PP set, and because the underlying seroconversion rates were expected to be 95% in each group.

The non-inferiority of a single dose of JE-CV compared with SA14–14–2, in terms of PRNT_50_ seroconversion rate^1^ 28 d after vaccination, was demonstrated in the PP set and confirmed in the FAS. All children except one (in Group SA14–14–2) were seroprotected^2^ 28 d after vaccination. GMTs had increased substantially in both groups; GMTRs (D28/D0) were slightly higher in Group JE-CV compared with Group SA14–14–2. Therefore, JE-CV is at least as immunogenic as SA14–14–2 vaccine after a single dose of either vaccine administered to healthy Korean children aged 12 to 24 mo. Both are live attenuated vaccines that share the same envelope antigens derived from the SA14 strain. The immunogenicity results of this study are consistent with those observed in earlier studies in toddlers vaccinated with JE-CV.^11,12,14^ Pre-existing immunity to JE did not impact the immune response induced by vaccination. Although the number of children with pre-existing anti-JE CV neutralizing antibodies is limited in this study (nine and 10 children in Group JE-CV and Group SA14–14–2, respectively), the results are in line with those obtained in previous clinical trials^11,12,14^ and suggest that JE-CV induces a protective immune response irrespective of the serological status against JE at baseline. The neutralizing antibody assessments used the JE-CV vaccine strain. In our prior experience the use of respective homologous vaccine strains to assess JE-CV and SA14–14–2 vaccine responses did not introduce any bias in the comparison of vaccine responses, perhaps because this strain contains the SA14 E protein common to both vaccines.^14^ In addition, JEV wild type is classified as a single serotype and JE-CV has been shown to induce neutralizing antibodies with comprehensive cross-coverage against isolates of the main genotypes of JEV wild type.^17^The safety of JE-CV has been evaluated in prospective clinical trials involving more than 5,000 subjects aged from 9 mo to 85 y. Double blind or observer blind comparisons with JE-VAX (MBDV), SA14–14–2 and placebo have shown a safety profile in favor of JE-CV.^10,14^ JE-CV was safe and well tolerated in the children who participated in this study, and there was no evidence to suggest that JE-CV is less safe than the SA14–14–2 vaccine that is licensed and used in the Republic of Korea and other countries in Asia. No fatal SAEs were reported, no child was withdrawn due to an AE and no unexpected reactions were observed. Only two SAEs (pyrexia in two children in Group SA14–14–2 that occurred within 2 d after vaccination) were assessed as related to vaccination; the origin of the fever was not determined. One child (in Group SA14–14–2) experienced a febrile convulsion 26 d after vaccination; this SAE was not related to vaccination as reported by the investigator and the child recovered fully. Events suggestive of hypersensitivity/allergic reactions (rhinitis allergic, rash and urticaria) were reported in few children; only two urticaria episodes (one child in each group) were assessed as related to vaccination by the investigator. No event suggestive of viscerotropic or neurotropic disease was reported. In both groups, only 4.4% of children experienced a severe solicited systemic reaction within 14 d after vaccination; for 10 out of 12 of these children, the severe systemic reaction was experienced simultaneously with unsolicited AEs (such as common cold, hand-foot-and-mouth disease, otitis media, bronchitis or pharyngitis) or SAEs (such as gastroenteritis, intussusception or fever) that were unrelated to vaccination and could be responsible for at least some of the solicited systemic AEs reported in these children. Most severe solicited systemic reactions were rated severe for no more than 3 d and resolved within 14 d after vaccination. Unsolicited AEs were mainly diseases of childhood (nasopharyngitis, diarrhea, fever and rhinorrhea) and most were unrelated to vaccination. The safety results of a single dose of JE-CV in this study are consistent the results observed in earlier studies in toddlers vaccinated with JE-CV.^11,12,14^

This is one of only two studies to compare the immunogenicity and safety of JE-CV with SA14–14–2 vaccine.^14^ Both studies have used the same methodology with a centralized laboratory for antibody testing and same procedures for AE collection. In the past 5 y, only studies with new generation inactivated vaccines have been undertaken with this level of reliability. The study was designed to demonstrate the non-inferiority of JE-CV compared with the SA14–14–2 vaccine (as required by Health Authorities for registration). However, larger groups may have revealed some potential differences between the two vaccines, especially in terms of immunogenicity.

At the time of initiating this study the schedule for JE-CV was a single dose. Subsequently, the need for a booster dose of JE-CV in children 12−24 mo after the primary vaccination (as approved now in all countries) was confirmed. Therefore, an open follow-up (booster) study has been initiated separately to give a booster dose of JE-CV to children who received the primary dose of JE-CV in this study; the results of the follow-up study are not yet available. The first comparative study of JE-CV and the SA14–14–2 vaccine in children provided immunogenicity and safety data up to 12 mo after vaccination.^14^

JE was the leading cause of viral encephalitis among children in the Republic of Korea in the 20th century, but since the 1980s JE disease has been on the decline after the implementation of a mass vaccination program.^18^ MBDV (Nakayama strain) was first introduced in Korea in 1971 but was not widely distributed until 1983, when an outbreak resulting in 1,197 cases and 40 deaths occurred.^18^ SA 14–14–2 vaccine has been licensed in Korea since 2002. MBDV (Nakayama strain) and SA 14–14–2 vaccine have been used as the National Immunization Program (NIP) vaccines since 1983 and 2013, respectively. The recommended schedule of MBDV in Korea is three doses for primary immunization starting at 12 mo of age with two booster doses at 6 y and 12 y. The Korea Centers for Disease Control and Prevention (KCDC) maintains the national notifiable disease surveillance system (NNDSS) and JE is listed as a national notifiable disease. There have been fewer than 30 cases of JE each year for the past 20 y in Korea. Thanks to many efforts, JE has been controlled despite being considered endemic in Korea.

JE-CV was found to be safe and well tolerated in the children who participated in our study. There is a persistent trend noted on the lower estimates of reactogenicity and other safety endpoints in the JE-CV group compared with the SA14–14–2 vaccine group; a similar observation was recently made in another comparative study in Thailand.^14^ JE-CV will help to protect children in JEV endemic areas with a good immunogenicity and safety profile.

## Conclusions

A single dose of JE-CV or SA14–14–2 vaccine elicited a comparable immune response with a good safety profile. All children except one (in Group SA14–14–2) were seroprotected 28 d after vaccination. JE-CV was well tolerated and no safety concerns were identified. The safety profile of JE-CV was found to be good. Immunogenicity and safety results in healthy Korean children aged 12 to 24 mo vaccinated with JE-CV are consistent with those obtained in previous studies conducted with JE-CV toddlers in other countries.

## Patients and Methods

### Study design

This was a randomized, observer-blind, controlled (SA14–14–2 vaccine), multicenter trial in children aged 12 to 24 mo conducted at 10 sites in the Republic of Korea. Children received one injection of a JE vaccine (either JE-CV or SA14–14–2 vaccine) on D0. To comply with the recommended immunization schedule for SA14–14–2 vaccine in Korea, booster doses of SA14–14–2 vaccine were to be given at 1 y after vaccination (i.e., outside the scope of this study). For children who received a first dose of JE-CV, a recommended booster dose 12 to 24 mo after primary immunization was to be offered in a separate clinical trial.

The study was conducted in accordance with the Declaration of Helsinki (version in force at the time of the study), Good Clinical Practice, International Conference on Harmonization guidelines, the European Directive 2001/20/EC and applicable national and local requirements. The protocol was approved by the Institutional Review Board of each study center and written informed consent was obtained from at least one parent or legally acceptable representative.

### Participants

We enrolled children in general good health without significant medical history into the study. Children were excluded if they fulfilled any of the following criteria: active, planned, or recent activity in another clinical study; receipt or planned receipt of antiviral medication within 2 mo or any vaccine within 4 wk before first trial vaccination and up to 4 wk after trial vaccination; receipt of any blood product in the preceding 3 mo that may interfere with assessment of immune response; immunodeficiency (any cause); history of central nervous system disorder or disease, including seizures; chronic illness that may interfere with trial conduct or completion; administration of systemic corticosteroids for more than 2 consecutive weeks within 4 wk preceding vaccination; febrile illness or moderate or severe acute illness or infection up to 3 d before vaccination; thrombocytopenia or bleeding disorder contraindicated for vaccination with the same administration route, or receipt of anti-coagulants within 3 wk of inclusion; known hypersensitivity to any vaccine components; history of flavivirus infection or vaccinations (including JE).

### Procedures

There were 2 visits (D0 and D28) and a phone call 6 mo after the D28 visit. After enrolment on D0, children were randomized 1:1 to receive one dose of JE-CV or SA14–14–2 vaccine (Group JE-CV or Group SA14–14–2, respectively). Treatment allocation was done using an interactive voice response system (IVRS). The randomization list was prepared by the sponsor biostatistics platform. Randomization was done using the permuted block method with stratification on study center. The investigator assigned an inclusion number (3-digit center identifier and 5-digit subject identifier corresponding to the chronological order of enrolment in the center) to each child. Each dose had a unique number and the vaccinator took one dose corresponding to the group assigned according to the inclusion number on the randomization list. Reconstituted JE-CV or SA14–14–2 vaccine was administered as a subcutaneous injection in the upper arm. The parents/representatives of children and the investigator observing safety were blinded to the vaccine administered. The vaccinator prepared and administered the vaccine in a separate room from where the observer-blind investigator assessed safety; the vaccinator did not collect any safety data. Blood samples for immunogenicity were taken on D0 (pre-vaccination) and D28. Children were observed for 30 min after vaccination to record any immediate AEs. Parents/representatives were given a digital thermometer for axillary temperature measurement, a ruler for measuring injection site reactions and a diary card for recording solicited and unsolicited AEs after vaccine administration.

### Vaccines

JE-CV was bulk manufactured in the US and lyophilized at Government Pharmaceutical Organization – Mérieux Biological Products (GPO-MBP), Thailand, and reconstituted using 0.4% sodium chloride diluent for injection; each 0.5mL dose contained 4.0−5.8 log_10_ plaque forming units (PFU) of virus. SA14–14–2 vaccine was manufactured by Chengdu Institute of Biological Products, People's Republic of China and purchased in Korea, and reconstituted using water for injection; each 0.5mL dose contained ≥ 5.4 log_10_ PFU of virus.

### Serology

JE neutralizing antibody levels were assessed by PRNT_50_ in Vero cells (Focus Diagnostics Inc.; Cypress, CA, US) as follows. Test serum and assay quality control serum samples were initially diluted at 1:5 in the first wells of 24-well plates followed by 2-fold serial dilutions up to 12 wells. JE-CV virus was diluted in cell culture media to yield 30−50 plaques per well in the virus control wells, and an equal volume of JE-CV virus added to each diluted serum sample. The virus-serum mixture was incubated at 2−8°C in 5% CO_2_ for 16−20 h to allow neutralization to occur and subsequently transferred to 24-well Vero cell seeded plates and incubated at 37°C in 5% CO_2_ for 1 h. One mL of methyl cellulose overlay medium was added to each well and plates incubated at 37°C in 5% CO_2_ for five days. After incubation, cells were fixed and stained using crystal violet-formaldehyde solution. The presence of JE-CV virus infected cells is indicated by the formation of viral plaques, visualized as clear spots on a violet background and counted. The neutralization titer (PRNT_50_) of the test serum sample is defined as the reciprocal of the highest test serum dilution for which the virus infectivity is reduced by 50% when compared with the average plaque count of the challenge virus control.^11^ The theoretical lower limit of quantitation (LLOQ) of the assay is a PRNT_50_ titer of 10 (reciprocal dilution). This assay was validated demonstrating inter-assay precision at ≤ 30% coefficient of variation (CV), and intra-assay precision at ≤ 20% CV for medium and high titer samples and ≤ 30% CV for low titer samples. Routine assay performance demonstrates variability within one 2-fold difference of median observed titers.

### Outcome measures

Seroconversion, defined as a JE neutralizing antibody titer ≥ 10 (1/dil) (which is the broadly accepted correlate of protection ^4,16^) in children who were seronegative (titer < 10 [1/dil]) at baseline or a ≥ 4-fold rise in JE neutralizing antibody titers in children who were seropositive (titer ≥ 10 [1/dil]) at baseline, was assessed on D28. GMTs and seroprotection rates, where seroprotection is defined as a JE neutralizing antibody titer ≥ 10 (1/dil), were assessed on D0 and D28. GMTRs (D28/D0) were also determined.

Safety endpoints included time to onset, number of days of occurrence and intensity of solicited (pre-listed in the child's diary and electronic case report form [eCRF]) injection site reactions (tenderness, erythema and swelling) up to 7 d after vaccination and solicited systemic reactions (fever, vomiting, crying abnormal, drowsiness, appetite lost and irritability) up to 14 d after vaccination. Unsolicited AEs up to 28 d after vaccination were collected including the time to onset, duration and intensity; ARs were unsolicited-AEs considered by the investigator to be related to vaccination. All solicited injection site and systemic reactions were considered to be related to vaccination by definition.

The following were denoted as AESIs for the JE-CV vaccine and collected up to 28 d after vaccination: hypersensitivity/allergic reactions, neurological events including febrile convulsions and vaccine failure. All SAEs were collected from Day 0 until 6 mo after vaccination. AEs were coded using the Medical Dictionary for Regulatory activities (MedDRA version 13.0) preferred term.

### Statistical methods

The statistical analysis was done using SAS® Version 9.2 software (SAS Institute Inc., Cary, NC, USA). A sample size of 300 children (150 children by group) was anticipated to allow 129 evaluable children in each group (projected attrition rate of 14%). This sample size was planned to provide a power of 90% in a one-sided test for non-inferiority at a significance level α of 0.025 and a 10% clinical non-inferiority margin when the underlying seroconversion rates were expected to be 95% in each group. The sample size for the immunogenicity analysis was calculated using the Farrington and Manning formula.^19^ For safety in the JE-CV group a sample size of 150 children vaccinated was planned to allow detecting with a 0.95 probability an AE with a frequency of 2%.

For the primary objective, a non-inferiority test was performed using the 95% 2-sided CI of the difference in D28 rate of seroconversion against the JE-CV virus between the JE-CV and SA14–14–2 vaccine groups. The 95% CI of the difference was calculated based on the Wilson score method without continuity correction.^20,21^ Non-inferiority was considered demonstrated if the lower limit of the 2-sided 95% CI was above −10%, as recommended.^15^ The analysis was performed on the PP set (children who were seronegative for JE at baseline and received a single dose of vaccine without any protocol deviations) and confirmed in the FAS of all children who were randomized and vaccinated, analyzed by vaccine group to which they were randomized.

Other immunogenicity and safety endpoints were analyzed descriptively. To provide GMTs/GMTRs and their 95% CIs, it was assumed that the log_10_ transformation of the titers/ratios followed a normal distribution, and the mean and 95% interval were calculated on log_10_ (titers/ratios) using the usual calculation for normal distribution, and then antilog transformations were applied to the results. The 95% CIs of percentages were computed using the exact binomial distribution.^21^ Immunogenicity analyses were performed on the PP set and FAS. Safety analyses were performed on the safety analysis set of all children who were vaccinated, analyzed by vaccine received.

A preliminary statistical analysis was performed on safety and immunogenicity data collected up to 28 d post-vaccine administration. The final analysis was performed at 6 mo after vaccination, on safety data collected at the 6-mo follow-up phone call.

## Trial Registration

ClinicalTrials.gov: NCT01396512
